# Convergent DNA methylation abnormalities at enhancers and bivalent promoters in human growth disorders

**DOI:** 10.1186/s13072-025-00650-1

**Published:** 2025-12-27

**Authors:** Marie E. S. Wheeler, Yoshiko Takahashi, Jihye Lee, Camille T. Perez, Xiaoting Chen, Yuri Lee, Zachary S. Pope, Daniella J. Lu, Marcus Seldin, Ivan Marazzi, Hongseok Yun, Matthew T. Weirauch, Minji Byun

**Affiliations:** 1 Department of Microbiology and Molecular Genetics, School of Medicine, University of California, Irvine, Irvine, CA USA; 2 Precision Immunology Institute, Icahn School of Medicine at Mount Sinai, New York, NY USA; 3 Center for Autoimmune Genomics and Etiology, Cincinnati Children’s Hospital Medical Center, Cincinnati, OH USA; 4 Department of Biological Chemistry, School of Medicine, University of California, Irvine, Irvine, CA USA; 5 Center for Epigenetics and Metabolism, School of Medicine, University of California, Irvine, Irvine CA, USA; 6 Department of Genomic Medicine, Seoul National University Hospital, Seoul, Republic of Korea; 7 Divisions of Allergy and Immunology, Biomedical Informatics, Human Genetics, and Developmental Biology, Cincinnati Children’s Hospital Medical Center, Cincinnati, OH USA; 8 Department of Pediatrics, University of Cincinnati College of Medicine, Cincinnati, OH USA

**Keywords:** Growth disorders, Overgrowth syndrome, DNMT3A, NSD1, DNA methylation, Bivalent chromatin

## Abstract

Loss-of-function mutations in *DNMT3A*, a DNA methyltransferase, or *NSD1*, a histone methyltransferase, cause overgrowth syndromes. Conversely, disruption of the DNMT3A domain that binds NSD1-deposited H3K36 dimethylation (H3K36me2) results in growth restriction. To investigate the molecular basis of these opposing growth outcomes, we generated isogenic human embryonic stem cells carrying growth syndrome–associated mutations in *DNMT3A* and *NSD1*. Unexpectedly, both overgrowth- and growth restriction–associated *DNMT3A* mutations led to DNA hypomethylation in a shared subset of active enhancers, implicating H3K36me2 in directing enhancer methylation maintenance. In contrast, bivalent promoters—marked by both active and repressive histone modifications—showed divergent DNA methylation changes: hypermethylation in growth restriction-associated *DNMT3A* mutants and hypomethylation in overgrowth-associated *DNMT3A* or *NSD1* loss-of-function mutants. These findings identify locus-specific DNA methylation defects as a common molecular feature and nominate dysregulated DNA methylation at bivalent promoters as a potential driver of abnormal growth phenotypes.

**Supplementary Information:**

The online version contains supplementary material available at 10.1186/s13072-025-00650-1.

## Introduction

*DNMT3A* encodes a de novo DNA methyltransferase essential for establishing DNA methylation patterns during development [1]. Germline mutations in *DNMT3A* underlie two distinct Mendelian syndromes with opposing growth phenotypes. Heterozygous loss-of-function (LoF) mutations cause Tatton-Brown-Rahman Syndrome (TBRS; OMIM 615879), characterized by tall stature, macrocephaly, intellectual disability, and dysmorphic features [2, 3]. Conversely, specific heterozygous missense mutations in the PWWP (Pro-Trp-Trp-Pro) domain have been linked to Heyn-Sproul-Jackson syndrome (HESJAS; OMIM 618724), marked by growth restriction, microcephaly, and impaired intellectual development [4, 5]. The PWWP domain recognizes di- or tri-methylated lysine 36 of histone H3 (H3K36me2/3) [6, 7], while the isoform-specific N-terminal region of DNMT3A1 binds monoubiquitinated lysine 119 of histone H2A (H2AK119ub1), directing DNMT3A to its genomic methylation targets [8, 9]. The catalytic methyltransferase domain, located near the C-terminus, is autoinhibited by the ADD (ATRX-Dnmt3-Dnmt3L) domain. When the ADD domain binds unmodified H3K4, it relieves autoinhibition and enables catalytic activity [10]. Together, these domains coordinate the recruitment and enzymatic activity of DNMT3A in a chromatin-context-dependent manner.

Intriguingly, mutations in other epigenetic regulators produce growth disorders with clinical features overlapping the *DNMT3A*-mutated syndromes TBRS and HESJAS [11]. For example, LoF mutations in *NSD1*, a histone methyltransferase that deposits H3K36me1/2 [12], cause Sotos syndrome (OMIM 117550), the most common overgrowth-intellectual disability syndrome with features that overlap with TBRS [11, 13–15]. Conversely, *NSD1* copy number gains due to microduplications are associated with microcephaly, short stature and developmental delay, clinical features similar to HESJAS [16, 17]. Similarly, LoF mutations in *EZH2*, *EED*, *SUZ12*–each encoding core components of the Polycomb Repressive Complex (PRC) 2 that deposits mono-, di- and trimethylation of lysine 27 on histone H3 (H3K27me1/2/3)–cause overgrowth-intellectual disability syndromes: Weaver syndrome (OMIM 277590), Cohen-Gibson syndrome (OMIM 617561), and Imagawa-Matsumoto syndrome (OMIM 618786), respectively [18–22].

Genome-wide DNA methylation studies have identified distinct DNA methylation abnormalities associated with the *DNMT3A*-mutated growth syndromes TBRS and HESJAS. Samples from overgrowth TBRS patients display focal hypomethylation at enhancers, Polycomb repressed regions, and developmental genes such as homeobox domains [23–25]. In contrast, samples from growth restriction HESJAS patients show hypermethylation at Polycomb-marked DNA methylation valleys and key developmental genes [4]. Despite these insights, differences in cell type, age, sex, and genetic background in patient-derived samples complicate direct comparisons across syndromes. Moreover, because these studies analyzed fully differentiated tissues such as blood, it remains unclear when during development the DNA methylation defects arise.

To address these limitations, we introduced TBRS- and HESJAS-associated *DNMT3A* mutations into human embryonic stem cells (hESCs), enabling assessment of their impact on DNA methylation in a controlled, isogenic, and early developmental context. To further contextualize *DNMT3A*-dependent changes, we also analyzed the DNA methylation profile of hESCs engineered to harbor *NSD1* LoF mutations associated with Sotos syndrome. We focused on Sotos syndrome among overgrowth-intellectual disability syndromes due to NSD1’s role in depositing H3K36me2, a histone modification recognized by the DNMT3A PWWP domain, and prior evidence of methylation similarities between Sotos syndrome and TBRS [6, 24, 26]. Together, these models provide a unique opportunity to dissect how DNMT3A activity is guided by histone modifications and how disruption of this interaction, through disease-associated mutations, contributes to abnormal growth phenotypes via altered DNA methylation landscapes.

## Results

### Generation of hESC models harboring DNMT3A and NSD1 mutations associated with human growth syndromes

**Fig. 1:**
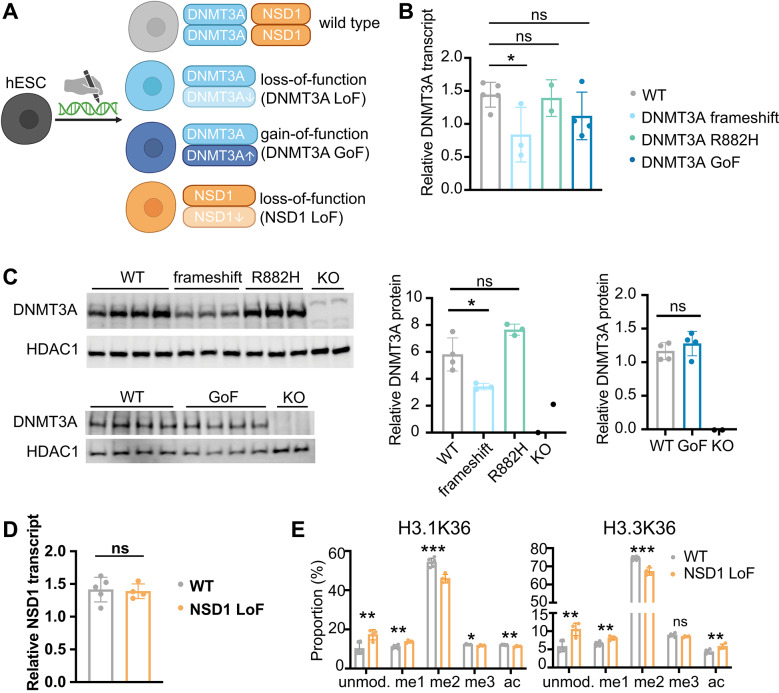
Generation and validation of hESC models for human growth syndromes. **A** Schematic of generating human embryonic stem cell (hESC) models that harbor heterozygous growth syndrome-associated mutations in *DNMT3A* and *NSD1* by CRISPR-Cas9 genome engineering. Blue indicates *DNMT3A* gene and orange indicates *NSD1* gene. Loss-of-function (LoF) mutations are indicated by a down arrow whereas DNMT3A gain-of-function (GoF) mutation is indicated by an up arrow. For the rest of the manuscript, all data points, bars, and lines are color-coded as follows: gray for wild type (WT), light blue for DNMT3A LoF, dark blue for DNMT3A GoF, and orange for NSD1 LoF. Schematic was created using BioRender. **B** Relative expression of DNMT3A transcripts as measured by real-time quantitative PCR, normalized to RNA18S transcript levels. Each dot represents an independent clone. **C** Western blot analysis of DNMT3A protein expression. See Table 1 for detailed genotypes of the mutant clones. DNMT3A knockout (KO) clones, generated previously [50], were included as controls. HDAC1 was used as a loading control. Each lane represents an independent clone. The plots on the right show the relative intensity of DNMT3A bands, normalized to HDAC1. **D** Relative expression of NSD1 transcripts as measured by real-time quantitative PCR, normalized to RNA18S transcript levels. See Table 1 for detailed genotypes of the NSD1 LoF clones. **E** Mass spectrometry of histones H3.1 and H3.3 K36 modifications in WT and NSD1 LoF hESCs. Each dot represents an independent clone. unmod., unmodified; me1, monomethylated; me2, dimethylated; me3, trimethylated; ac, acetylated. For panels (**B**, **C**, **D**, and **E**), statistical significance was determined by Student’s t-test. *p < 0.05; **p < 0.01; ***p < 0.001; ns, not significant. Bar graphs show the mean ± standard deviation.

To isolate mutation-intrinsic effects from inter-individual genetic and epigenetic variability, we introduced growth syndrome-associated mutations in *DNMT3A* and *NSD1* into an isogenic H1 (WA01) hESC line (Fig. 1A). To model TBRS, we introduced heterozygous *DNMT3A* frameshift mutations that produce premature stop codons, or the heterozygous R882H mutation, which functions in a dominant-negative manner [27, 28]. We refer to these collectively as DNMT3A LoF mutants. To model HESJAS, we introduced previously reported heterozygous W330R or D333N missense mutations in *DNMT3A* [4]. We refer to these as DNMT3A gain-of-function (GoF) mutants. For Sotos syndrome, we introduced heterozygous *NSD1* frameshift mutations that produce premature stop codons, referred to as NSD1 LoF mutants (Table 1, Supplemental Table 1, Supplemental Fig. 1A). The parental cell line as well as three clones that had undergone similar targeting and selection processes without acquiring mutations in the targeted loci were used as wild type (WT) controls throughout the study.

**Table 1:** Genotype of hESC mutant clones

Category	Clone number	Gene	cDNA	protein	Model for:
DNMT3A frameshift	1	DNMT3A	WT/c.791_792insA	WT/p.V265Rfs*16	Tatton-Brown-Rahman syndrome
	2	DNMT3A	WT/c.791_792insTGA GC	WT/p.V265Efs*53	
	3	DNMT3A	WT/c.989_1007del	WT/p.W330Sfs*9	
DNMT3A R882H	1	DNMT3A	WT/c.2645G > A	WT/p.R882H	
	2	DNMT3A	WT/c.2645G > A	WT/p.R882H	
DNMT3A GoF	1	DNMT3A	WT/c.988 T > C	WT/p.W330R	Heyn-Sproul-Jackson syndrome
	2	DNMT3A	WT/c.988 T > C	WT/p.W330R	
	3	DNMT3A	WT/c.997G > A	WT/p.D333N	
	4	DNMT3A	WT/c.997G > A	WT/p.D333N	
NSD1 LoF	1	NSD1	WT/c.1093_1094insT	WT/p.Y365Lfs*10	Sotos syndrome
	2	NSD1	WT/c.1092delC	WT/p.Y365Tfs*54	
	3	NSD1	WT/c.1092_1094delC TAinsTAGGGCCTAT	WT/p.Y365Rfs*12	
	4	NSD1	WT/c.1091_1097delA CTACGT	WT/p.Y364Wfs*53	

DNMT3A cDNA and protein coordinates are based on NM_175629.2 and NP_783328.1, respectively. NSD1 cDNA and protein coordinates are based on NM_022455 and NP_071900, respectively

Expression levels of pluripotency markers including *POU5F1* and *SOX2* were comparable between mutant and WT clones (Supplemental Fig. 1B and C), indicating that the *DNMT3A* and *NSD1* mutations do not significantly impact hESC pluripotency under standard culture conditions. DNMT3A frameshift clones exhibited reduced mRNA and protein levels of DNMT3A compared to WT controls, presumably due to nonsense-mediated mRNA decay triggered by premature stop codons (Fig. 1B and C). In contrast, DNMT3A R882H and GoF mutants maintained mRNA and protein levels of DNMT3A comparable to those in WT clones, consistent with previous reports indicating functional impairment without loss of expression [4, 28, 29] (Fig. 1B and C). Although NSD1 LoF mutations are predicted to introduce premature stop codons, *NSD1* mRNA levels in NSD1 LoF clones were not significantly reduced relative to those in WT controls (Fig. 1D). To validate NSD1 LoF mutations functionally, we analyzed relative abundance of modified H3K36 via mass spectrometry. NSD1 LoF mutants displayed a significant reduction in dimethylated H3K36, with a corresponding increase in unmodified and monomethylated forms at both replication-coupled H3.1 and replication-independent H3.3 histone H3 variants [30, 31] (Fig. 1E). These results are consistent with the reduction in H3K36me2 observed in *Nsd1*-depleted mouse ESCs [6] and confirm that NSD1 LoF hESC clones are functionally impaired, despite retaining normal *NSD1* transcript levels.

### Growth syndrome-associated DNMT3A and NSD1 mutations cause DNA methylation defects in hESCs

**Fig. 2:**
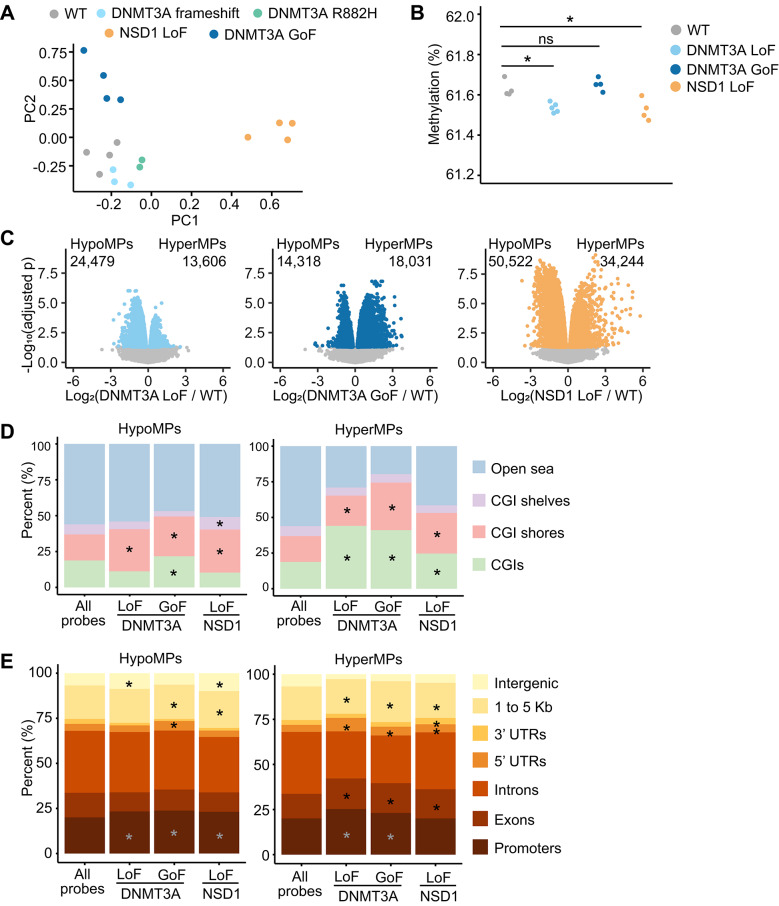
Mutant hESCs display DNA methylation defects. **A** Multidimensional scaling of principal components (PCs) of the top 10,000 differentially methylated positions. **B** Mean methylation (EPIC probe beta values expressed as a percentage) across all analyzed EPIC probes (n = 818,992). Each dot represents an independent clone. Statistical significance was determined by Student’s t-test. *p < 0.05; ns, not significant. DNMT3A frameshift and R882H clones are grouped together under the DNMT3A LoF category. **C** Volcano plots of the log_2_ fold change of the methylation values of mutant to WT plotted against the negative log_10_ of the adjusted p value. Each dot represents a probed CpG site. Colored dots, other than gray, represent significantly hypomethylated positions (HypoMPs) or hypermethylated positions (HyperMPs) with adjusted p < 0.05. The numbers of significantly differentially methylated positions are indicated. **D** Distribution of HypoMPs and HyperMPs relative to CpG island (CGI) regions expressed as a percentage of probes within each category. CGI annotations, including CGI shores, CGI shelves, and open sea follow the definitions in the annotatr package [60]: CGI shores are defined as the 2 kb regions flanking the CGI boundaries; CGI shelves are defined as the 2 kb regions immediately upstream and downstream of the CGI shores, on the side opposite the CGIs. Statistical significance was determined by Fisher’s test. *p < 0.05. **E** Distribution of HypoMPs and HyperMPs relative to genes expressed as a percentage of probes within each category. Annotations follow the definitions in the annotatr package for basic genes and intergenic regions: 1 to 5 Kb are defined as 1–5 Kb upstream of a transcriptional start site and promoters as < 1 Kb upstream of a transcriptional start site. UTR, untranslated regions of genes; Kb, kilobases. Statistical significance was determined by Fisher’s test. *p < 0.05

We assessed DNA methylation of passage-matched mutant and WT hESCs using the Infinium MethylationEPIC BeadChip array (EPIC). After normalization and filtering, 818,992 CpG probes remained for analysis (see Methods for details). Multidimensional scaling analysis of DNA methylation profiles separated clones according to mutation type, supporting the quality and consistency of our hESC models (Fig. 2A). Notably, DNMT3A frameshift and R882H mutants clustered closely and showed substantial overlap in differentially methylated CpG positions (DMPs) (Fig. 2A and Supplemental Fig. 2A). Furthermore, pairwise comparison of DMPs between frameshift and R882H clones revealed a near 1:1 correlation (Supplemental Fig. 2B), indicating highly similar DNA methylation defects. Conversely, pairwise comparisons between these clones and DNMT3A GoF or NSD1 LoF showed weaker or partial correlations (Supplemental Fig. 2C–F). Given their shared DNA methylation phenotype and the common clinical presentation of TBRS, we grouped frameshift and R882H mutants together under the DNMT3A LoF category for downstream analyses.

The average DNA methylation levels across all analyzed CpG positions showed a small but statistically significant reduction in DNMT3A LoF and NSD1 LoF clones (Fig. 2B). In agreement with these results, the DMP analysis revealed a greater number of hypomethylated positions (HypoMPs) compared to hypermethylated positions (HyperMPs) in these clones (Fig. 2C). In contrast, DNMT3A GoF clones exhibited more HyperMPs than HypoMPs, despite their overall average DNA methylation not significantly differing from WT controls (Fig. 2B and C). To further characterize these differences, we examined the genomic distribution of DMPs relative to CpG islands (CGIs) (Fig. 2D). HypoMPs across all mutant categories showed enrichment at CGI shores, while HyperMPs were enriched with both CGIs and their shores. Further analysis of DMP distribution showed consistent enrichment of HypoMPs within promoters across all clones, while HyperMPs were enriched 1–5 kb upstream of transcription start sites, in 5′ untranslated regions, and in exons (Fig. 2E). Notably, DNMT3A GoF clones showed enrichment of both HypoMPs and HyperMPs in these same regions, underscoring the need for more detailed characterization. Nevertheless, these significant methylation changes at CGIs, promoters, and their vicinity suggested that we are capturing biologically meaningful DNA methylation changes.

To further characterize the DMPs, we performed a Regulatory Element Locus Intersection (RELI) analysis [32] by intersecting HypoMPs and HyperMPs from each mutant group with over 10,000 publicly available occupancy datasets across diverse human cell types. The resulting enrichments are provided in Supplemental Table 2 as a reference framework that complements the focused analyses presented in the main text.

### DNA methylation defects in mutant hESCs mirror those observed in fully differentiated patient cells

We next examined whether the DNA methylation defects in mutant hESC lines reflect those present in patient samples. DNA methylation data from blood of individuals with DNMT3A- or NSD1-associated growth syndromes, generated using the Infinium HumanMethylation450K or EPIC arrays, were obtained from published studies [4, 23, 33] (Supplemental Table 3). To compare these datasets, we annotated each DMP using a chromatin state model called ChromHMM, which classifies genomic regions based on shared histone modification patterns, enabling genome-wide analysis with reduced dimensionality [34, 35]. To be able to compare our hESC data to patient methylation data from blood samples, we used a tissue-independent full-stack chromatin state annotation. This framework defines a set of broad chromatin states and further subdivides them into cell type–specific substates, resulting in a total of 100 distinct chromatin states [35].

**Fig. 3:**
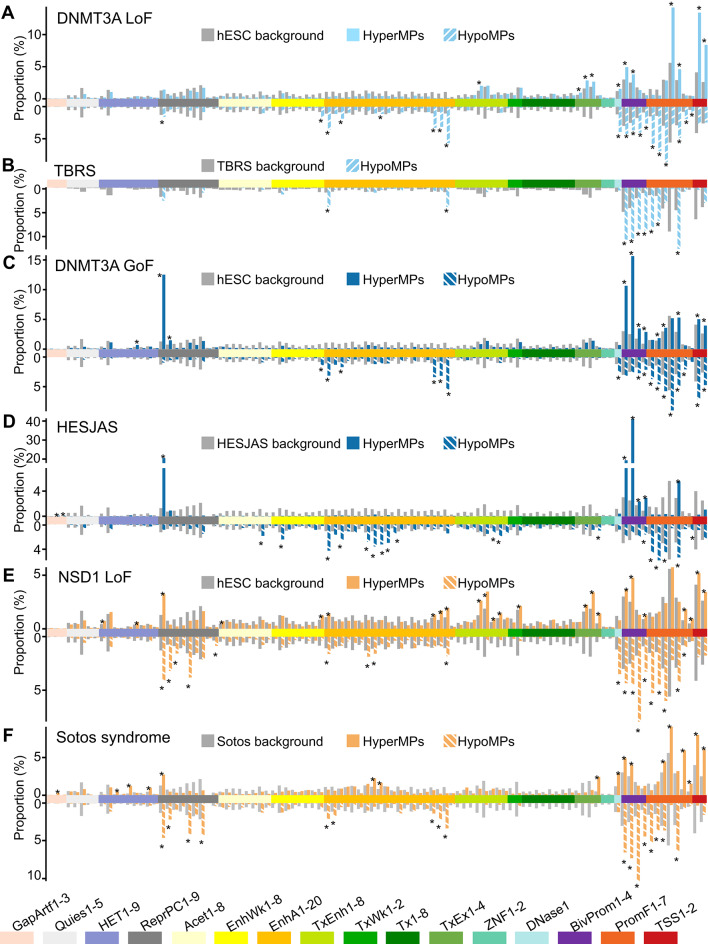
Chromatin state analysis of differentially methylated positions in hESC mutants and patient blood. Proportions of probes in each full-stack chromatin state for (**A**) DNMT3A LoF hESCs (HypoMPs n = 24,479; HyperMPs n = 13,606), (**B**) TBRS patient blood (HypoMPs n = 832), (**C**) DNMT3A GoF hESCs (HypoMPs n = 14,318; HyperMPs n = 18,031), (**D**) HESJAS patient blood (HypoMPs n = 2,796; HyperMPs n = 9,576), (**E**) NSD1 LoF hESCs (HypoMPs n = 50,522; HyperMPs n = 34,244), and (**F**) Sotos syndrome patient blood (HypoMPs n = 24,148; HyperMPs n = 4,168) Background represents the proportion of all probes analyzed in each state. Backgrounds differ across patient types because each dataset was processed separately, reflecting differences in patient characteristics and array platforms (see Methods). HyperMPs of TBRS patients were not analyzed due to their small size (n = 38). Statistical significance was determined by Fisher’s test. *p < 0.001. Legend of chromatin states [35]: GapArtf, assembly gaps and alignment artifacts; Quies, quiescent (low histone emissions, except possibly weak H3K9me3); HET, heterochromatin (H3K9me3); ReprPC, repressed polycomb (H3K27me3); Acet, various acetylations (weaker H3K4me1/2/3, H3K9ac, and H3K27ac emissions); EnhWk, weak enhancers; EnhA, active enhancers (enhancers marked by H3K4me1, DNase, H2A.Z, and/or H3K27ac); TxEnh, transcribed candidate enhancers; TxWk, weak transcription; Tx, strong transcription; TxEx, transcription and exons (marked by H3K36me3, H3K79me1, H3K79me2, or H4K20me1); ZNF, zinc finger (H3K36me3 and H3K9me3); DNase1, only DNase 1 hypersensitivity; BivProm, bivalent promoter (H3K27me3 and promoter marks); PromF, promoter flanking (H3K4me1 and promoter marks); TSS, transcriptional start sites (promoter marks of H3K4me2/3 and H3K9ac)

The predominant DMP category of each hESC mutant (HypoMPs for DNMT3A LoF and NSD1 LoF, and HyperMPs for DNMT3A GoF) displayed chromatin state enrichment patterns strikingly similar to those observed in the matching DMP category of corresponding patient blood samples (Fig. 3). HypoMPs from DNMT3A LoF hESCs and TBRS patient blood were significantly enriched in active enhancer (EnhA), bivalent promoter (BivProm), and promoter-flanking (PromF) chromatin states (Fig. 3A and B), while repressed Polycomb (ReprPC) and bivalent promoter states were enriched in HyperMPs from DNMT3A GoF hESCs and HESJAS patient blood (Fig. 3C and D). HypoMPs of NSD1 LoF hESCs and Sotos syndrome patient blood showed similar enrichment patterns compared to those in DNMT3A LoF hESCs and TBRS blood, with an additional enrichment in repressed Polycomb states (Fig. 3E and F). Together, these results demonstrate that disease-associated mutations induce DNA methylation defects already in undifferentiated pluripotent stem cells. Moreover, the hESC models faithfully recapitulate the chromatin-state categories disrupted in patient cells, indicating that enhancers, bivalent promoters, and Polycomb-repressed regions are preferentially affected across both pluripotent and differentiated contexts, even though the precise genomic loci differ between cell types.

### H2AK119ub1-marked active enhancers are hypomethylated in both DNMT3A LoF and GoF mutants, revealing a role for the PWWP-H3K36me2/3 interaction in maintaining DNA methylation at these regions

Although fewer than HyperMPs, DNMT3A GoF hESC mutants as well as HESJAS patient blood exhibit a substantial number of HypoMPs (Fig. 2C, 3C and D). We were intrigued by the highly similar chromatin state enrichment pattern of DNMT3A GoF HypoMPs and that of DNMT3A LoF HypoMPs (Fig. 3A and C). While the hypomethylation phenotype of DNMT3A LoF and TBRS patients has been extensively studied [23–25, 36], previous studies of DNMT3A GoF mutations have focused mainly on hypermethylated regions with limited characterization of hypomethylated ones [4, 37].

**Fig. 4:**
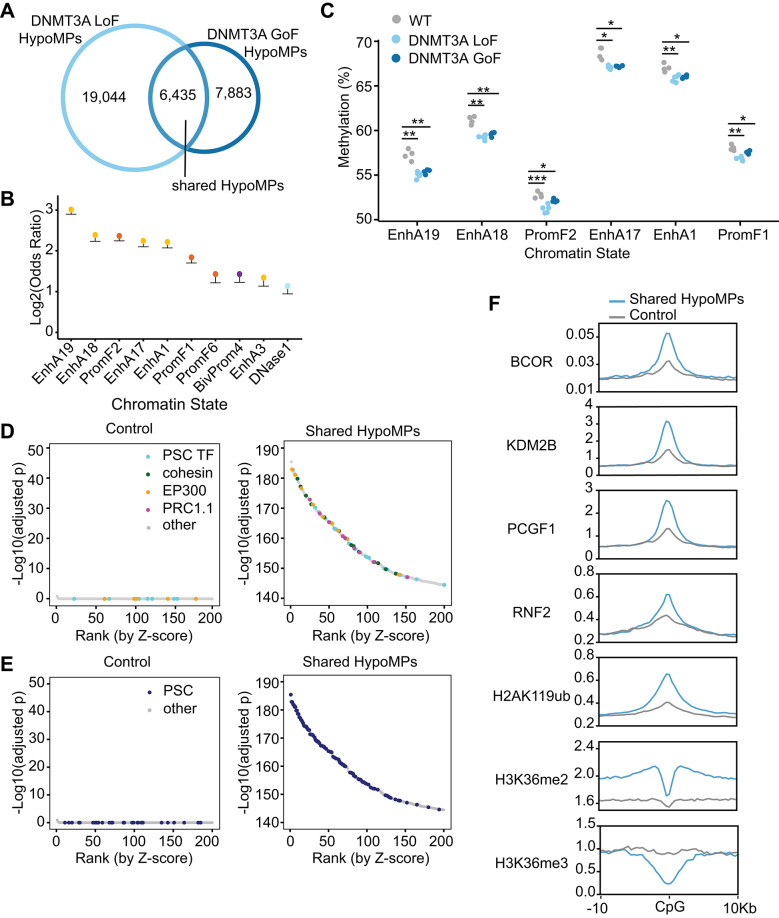
Shared hypomethylation of H2AK119ub1-marked active enhancers in DNMT3A GoF and LoF mutants. **A** Number of overlapping HypoMPs between DNMT3A LoF and DNMT3A GoF mutants. P < 2.2*10^–16^, Fisher’s exact test of unique DNMT3A GoF in all probes compared to DNMT3A GoF shared with DNMT3A LoF. **B** Log_2_ odds ratios showing enrichment of shared HypoMPs from DNMT3A mutants across full-stack chromatin states. Dots are colored according to the chromatin state category colors shown in Fig. 3, with lower confidence interval bounds indicated. Upper confidence intervals extended to positive infinity, reflecting uncertainty due to small sample sizes, and are omitted for clarity. Top 10 enriched states among 100 total states are shown (all p < 1*10^–18^). **C** Mean DNA methylation values at CpG positions within selected enhancer chromatin states. Each dot represents an independent clone. Statistical significance was determined by Student’s t-test. *p < 0.05; **p < 0.01; ***p < 0.001. **D**, **E** RELI analysis of shared HypoMPs intersected with > 10,000 public chromatin datasets. Shown are the top 200 datasets ranked by Z-score. Most enriched chromatin factors (**D**) and cell types (**E**) are highlighted. PSC TF: POU5F1, NANOG, SOX2, cohesion: RAD21, NIPBL, PRC1.1: BCOR, KDM2B, PCGF1, RYBP, RNF2. Controls represent randomly sampled EPIC probe regions. Each dot represents a dataset profiling a chromatin factor in a human cell type. Supplemental Table 2 contains RELI results of all examined datasets. PSC, pluripotent stem cells; TF, transcription factors. **F** CUT&RUN (H2AK119ub1) or ChIP-seq (all others) signal, averaged in 400-bp bins, across a 20-kb window (± 10 kb) centered on shared HypoMPs or matched control regions in WT hESCs. The y-axis indicates relative signal intensity normalized as described in the Methods section or in the original studies. BCOR, KDM2B, PCGF1, H3K36me2 occupancy data are from GEO accession number GSE104690, RNF2 data is from GSE105028, H3K36me3 is from ENCODE (ENCSR476KTK), and H2AK119ub1 is from GSE301386 (this study).

DNMT3A GoF mutations in HESJAS disrupt the PWWP domain’s ability to bind H3K36me2/3 without affecting overall protein levels [4, 7, 38] (Fig. 1C), whereas DNMT3A LoF mutations broadly diminish the availability of catalytically active DNMT3A at its methylation targets either via reduced protein levels (frameshift) or impaired catalytic activity (R882H). Thus, we hypothesized that HypoMPs shared between DNMT3A GoF and DNMT3A LoF mutants represent genomic regions whose methylation relies on the DNMT3A PWWP-H3K36me2/3 interaction. To identify such regions, we intersected HypoMPs from DNMT3A LoF mutants with those from DNMT3A GoF mutants and analyzed chromatin states enriched at these shared sites (shared HypoMPs) (Fig. 4A).

The shared HypoMPs were significantly enriched in several enhancer and promoter-flanking chromatin states (Fig. 4B). Consistently, a pronounced reduction in DNA methylation was observed in enhancer states EnhA1,17,18, and 19 in both DNMT3A LoF and DNMT3A GoF mutants (Fig. 4C). For comparison, we also intersected HyperMPs from DNMT3A LoF and DNMT3A GoF mutants, which showed limited and inconsistent enrichment in transcription start site chromatin states (Supplemental Fig. 3A–C), further underscoring enhancer hypomethylation as the dominant signature of shared HypoMPs. While EnhA1 corresponds to enhancers broadly active across cell types, EnhA17-19 are specific to pluripotent stem cells including hESCs [35]. Interestingly, HypoMPs in the HESJAS patient’s blood were more strongly enriched in blood lineage-specific enhancer states (EnhA7-11) than in pluripotency-specific ones (Fig. 3D). Together, these findings suggest that the DNMT3A PWWP domain is required for maintaining DNA methylation at enhancers active in the corresponding cell type.

Further characterization of shared HypoMPs with RELI revealed highly significant overlap between shared HypoMPs and chromatin regions occupied by pluripotency-associated transcription factors (e.g., POU5F1, NANOG, SOX2), cohesion complex and loader complex components (e.g. RAD21, NIPBL), and transcriptional coactivators, particularly EP300 (Fig. 4D, Supplemental Table 4). Notably, the vast majority of the most significantly overlapping datasets were generated in pluripotent stem cells (Fig. 4E, Supplemental Table 4). Together, these results independently validate that shared HypoMPs are enriched in pluripotency-specific active enhancers.

RELI also uncovered significant overlap between shared HypoMPs and genomic regions occupied by PRC1.1 components, including BCOR, KDM2B, PCGF1, and RNF2 (Fig. 4D and F). PRC1.1 is a noncanonical variant of PRC1 that deposits H2AK119ub1 [39], and accordingly, we observed H2AK119ub1 enrichment at shared HypoMPs compared to control CpG sites (Fig. 4F). This was unexpected, as previous studies have shown that DNMT3A GoF mutant proteins, which lack the ability to bind H3K36me2/3, are redirected to H2AK119ub1-marked regions via the N-terminal ubiquitin-dependent recruitment motif, resulting in hypermethylation [8, 38]. Our data indicate that a subset of H2AK119ub1-marked regions, particularly enhancers embedded in environments enriched for H3K36me2 but not H3K36me3, are hypomethylated in DNMT3A mutants (Fig. 4F). This suggests that recognition of H3K36me2 by the DNMT3A PWWP domain is required for proper DNA methylation at these sites.

### Mutations associated with overgrowth versus growth restriction cause contrasting DNA methylation changes at bivalent chromatin

While hypomethylation of enhancers is a prominent feature in DNMT3A LoF mutants and TBRS, the overlap with DNMT3A GoF mutants and an HESJAS patient with growth restriction indicates that these shared enhancer changes may not be the primary drivers of the divergent growth phenotypes. To investigate alternative DNA methylation signatures potentially related to growth regulation, we searched for genomic regions with shared methylation changes in the two overgrowth-associated conditions—DNMT3A LoF and NSD1 LoF—but with opposing methylation changes in DNMT3A GoF mutants.

**Fig. 5:**
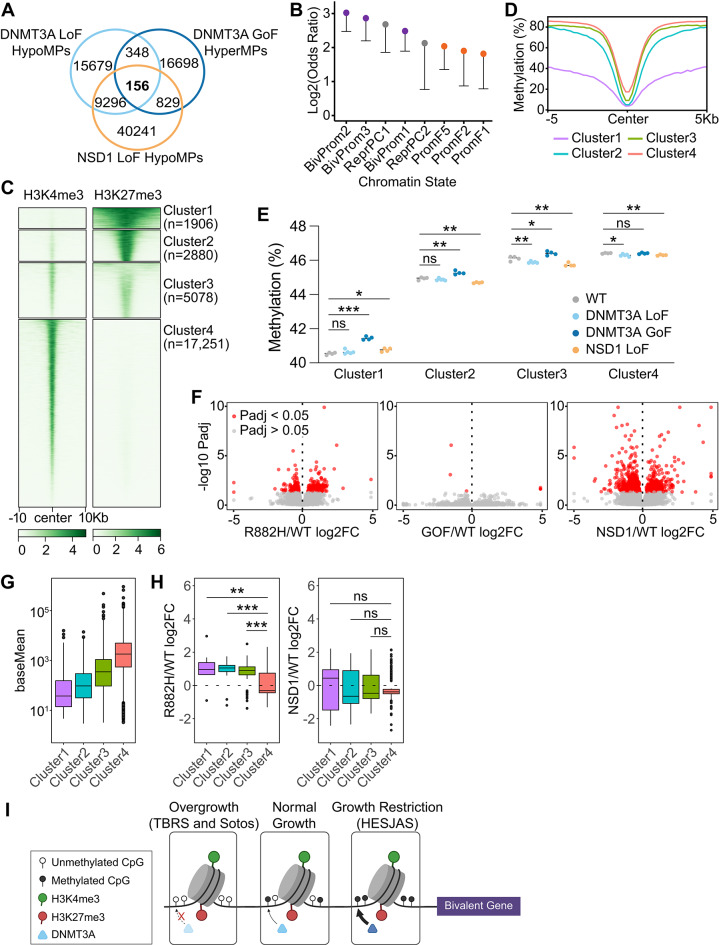
Contrasting DNA methylation changes at bivalent chromatin in overgrowth versus growth restriction hESC models. **A** Venn Diagram depicting the overlap of DNMT3A LoF HypoMPs with NSD1 LoF HypoMPs and DNMT3A GoF HyperMPs. **B** Log_2_ odds ratios showing enrichment of the 156 CpG sites across full-stack chromatin states. Dots are colored according to the chromatin state category colors shown in Fig. 3 and lower confidence interval bound is shown. Upper confidence intervals extend to positive infinity, reflecting uncertainty due to small sample sizes, and are omitted for clarity. States with significant enrichment (p < 0.05) are shown. **C** Heatmap of H3K4me3 and H3K27me3 signals at H3K4me3-marked genomic regions, clustered by H3K27me3 signal intensity in WT H1 hESCs. The number of regions in each cluster is indicated. Color scale indicates normalized signal intensity. See Methods for details. H3K4me3 and H3K27me3 CUT&RUN data were generated in-house (GEO accession number GSE301386). **D** DNA methylation profiles across cluster midpoints (± 5 kb) for the four clusters defined in panel C. **E** Mean DNA methylation values of CpG positions within clusters from panel C. Each dot represents an independent clone. Statistical significance was determined by Student’s t-test. *p < 0.05; **p < 0.01; ***p < 0.001; ns, not significant. **F** Differential gene expression in DNMT3A R882H, DNMT3A GoF, and NSD1 LoF mutants versus WT, shown as RNA-seq volcano plots. Genes with adjusted P (Padj) < 0.05 are highlighted in red. **G** RNA-seq baseline expression (baseMean: average normalized read counts) of genes across Clusters 1–4, illustrating the inverse correlation with H3K27me3 levels. **H** RNA-seq log₂ fold-change of DEGs in DNMT3A R882H vs. WT and NSD1 LoF vs. WT, grouped by clusters defined in panel C. Statistical significance was determined by Student’s t-test. **p < 0.01; ***p < 0.001; ns, not significant. **I** Model summarizing DNA methylation changes at bivalent promoters marked by H3K4me3 and H3K27me3 in *DNMT3A*- and *NSD1*-mutated growth syndromes. Reduced bivalent promoter methylation is associated with overgrowth phenotypes in TBRS and Sotos syndrome, whereas increased bivalent promoter methylation is associated with growth restriction in HESJAS.

Direct CpG-level comparison identified 156 CpG sites that were significantly hypomethylated in both DNMT3A LoF and NSD1 LoF mutants and hypermethylated in DNMT3A GoF mutants, with no CpGs exhibiting the reverse pattern (Fig. 5A, Supplemental Fig. 4A). These 156 CpG sites were strongly enriched for bivalent promoter states (Fig. 5B, Supplemental Table 5). Moreover, we noted that bivalent promoter states were markedly enriched among HyperMPs in DNMT3A GoF mutants, while enriched among HypoMPs in DNMT3A LoF and NSD1 LoF mutants (Fig. 3A, C, E). Importantly, these chromatin-state-specific patterns were mirrored in patient blood samples: bivalent promoter states were hypermethylated in the growth-restricted HESJAS patient and hypomethylated in the overgrowth syndromes TBRS and Sotos patients (Fig. 3B, D, F).

Bivalent promoters, defined by co-occupancy of H3K4me3 and H3K27me3, are typically associated with developmental genes that remain transcriptionally poised but are expressed at low levels in pluripotent stem cells [40]. To characterize these regions further, we clustered H3K4me3-marked regions in WT hESCs based on their H3K27me3 signal (Fig. 5C), identifying three bivalent clusters (Clusters 1–3) and an H3K4me3-only cluster (Cluster 4). These clusters aligned with bivalent promoter classes previously defined in hESCs [49]: Cluster 1 was enriched for high H3K27me3/H3K4me3 ratio (hiBiv) promoters, whereas Clusters 2 and 3 were enriched for low H3K27me3/H3K4me3 ratio (loBiv) promoters (Supplemental Fig. 4B, Supplemental Table 6). Within the bivalent clusters, H3K27me3 levels were inversely correlated with both H3K4me3 occupancy and DNA methylation (Fig. 5C and D). We also observed a positive correlation between H3K27me3 signal strength and the extent of DNA hypermethylation in DNMT3A GoF mutants (Fig. 5E). Among the bivalent clusters, only Cluster 3—characterized by relatively weak levels of both H3K4me3 and H3K27me3—showed significant hypomethylation in both DNMT3A LoF and NSD1 LoF mutants, as assessed by comparing mean methylation levels across all CpG sites within each cluster (Fig. 5E).

To assess whether altered DNA methylation at bivalent promoters is accompanied by transcriptional changes, we performed RNA-seq in mutant and WT hESCs. DNMT3A LoF (R882H) and NSD1 LoF cells exhibited several hundred differentially expressed genes (DEGs; adjusted p < 0.05), whereas DNMT3A GoF mutants displayed very few (Fig. 5F). Across Clusters 1–4, defined in Fig. 5C, baseline gene expression levels were inversely correlated with H3K27me3 signal (Fig. 5G). In DNMT3A R882H cells, DEGs mapping to bivalent clusters were more often upregulated than downregulated, a pattern not observed in NSD1 LoF cells (Fig. 5H). The limited number of DEGs in DNMT3A GoF cells precluded such analysis. Together, these results indicate that DNA methylation changes at bivalent promoters are linked to transcriptional effects in DNMT3A LoF mutants, whereas such effects are less evident in DNMT3A GoF and NSD1 LoF cells.

Together, these analyses reveal that bivalent promoters are differentially methylated in DNMT3A and NSD1 mutant hESCs in a manner consistent with their associated growth phenotypes: hypomethylation in DNMT3A LoF and NSD1 LoF mutants versus hypermethylation in DNMT3A GoF mutants (Fig. 5I). While RNA-seq in undifferentiated hESCs showed limited transcriptional changes corresponding to these methylation patterns, further studies in differentiated cell types will be necessary to comprehensively assess how these DNA methylation changes influence gene expression and developmental programs, and ultimately contribute to the divergent growth phenotypes.

## Discussion

In this study, we compared shared and distinct molecular defects in hESCs engineered with *DNMT3A* and *NSD1* mutations that underlie overgrowth and growth restriction syndromes. Our models recapitulate DNA methylation changes observed in patient blood samples, as confirmed by reanalysis of publicly available datasets, which is consistent with published interpretations [4, 23–25, 33, 36, 37]. These similarities underscore the utility of hESCs as a model system for investigating the molecular pathology of these disorders.

Interestingly, the reduction in H3K36me2 in NSD1 LoF hESCs was less than 50%, suggesting compensation by related histone methyltransferases such as NSD2 and NSD3, as recently shown [41]. Likewise, DNMT3A haploinsufficiency does not halve DNA methylation, likely due to both overlapping activity from DNMT1 and DNMT3B and the possibility that DNMT3A protein levels from a single functional allele are sufficient to maintain DNA methylation at most loci. Despite these compensatory mechanisms and near-normal global modification levels, disease still develops, indicating that specific genomic regions are critically dependent on the full dosage of *NSD1* or *DNMT3A*.

By introducing mutations into an isogenic background, we were able to directly compare mutation-specific effects without the confounding variables present in patient samples. This approach revealed an unexpected finding: shared hypomethylation at H2AK119ub1-marked active enhancers in both DNMT3A GoF and LoF mutants. This suggests a cooperative role of H2AK119ub1 and H3K36me2 in recruiting DNMT3A to active enhancers, and not just a competitive one [38]. Although this shared enhancer hypomethylation phenotype is unlikely to explain the divergent growth outcomes in TBRS and HESJAS, its functional relevance warrants further investigation, as it may contribute to other syndromic features associated with these disorders.

The contrasting DNA methylation defects observed in overgrowth versus growth restriction mutants implicate bivalent chromatin as a potential regulatory hub in organismal growth. Our RNA-seq analyses in undifferentiated hESCs revealed that DNMT3A LoF mutants show upregulation of genes associated with bivalent promoters, consistent with promoter hypomethylation. In contrast, NSD1 LoF mutants, while exhibiting transcriptional changes overall, do not show the expected upregulation of bivalent genes, and DNMT3A GoF mutants display few transcriptional changes despite hypermethylation of bivalent promoters. One possible explanation is that in DNMT3A GoF mutants, transcriptional repression of bivalent genes may be difficult to detect because they are generally transcriptionally inactive in hESCs. Conversely, NSD1 has been reported to have a catalytic-independent transcriptional coactivator function [42], such that its loss may affect transcription through mechanisms not directly reflected by DNA methylation. These findings underscore the need for future studies in differentiated cell types and patient samples—similar to recent work on Sotos syndrome patients [33]—to more comprehensively evaluate how these methylation changes impact transcriptional programs and contribute to divergent growth phenotypes in *DNMT3A*- and *NSD1*-mutated disorders.

Regulation of bivalent chromatin, particularly through modulation of PRC2 activity and H3K27me3 levels, appears to play a broader role in other overgrowth and growth restriction syndromes. For example, partial LoF mutations in EZH2, the catalytic subunit of PRC2, are associated with Weaver syndrome, the second most prevalent overgrowth-intellectual disability syndrome [11, 19]. These mutations have been shown to impair histone methyltransferase activity and reduce methylated H3K27 [43–45]. Conversely, a missense EZH2 mutation p.A738T, which enhances its catalytic activity [46, 47], has been reported in a patient with growth restriction [46], suggesting that the levels of H3K27me3 modulate growth outcomes in a dosage-dependent manner. Given the documented crosstalk among DNMT3A, NSD1, and PRC2 [48], it would be informative to compare gene expression changes across these models to identify shared or divergent regulatory consequences.

Our analysis of bivalent promoters provides initial insight into how PRC2-mediated regulation intersects with DNA methylation in these disorders. Our bivalent clusters can be related to bivalent gene classes described in mouse and human ESCs [49] (Supplemental Fig. 4B). These bivalent promoters have been shown to exhibit reductions in H3K27me3 upon DNA hypomethylation, transcriptional derepression during differentiation, and susceptibility to DNA hypermethylation in human cancer, with promoters having higher H3K27me3/H3K4me3 ratios demonstrating more pronounced effects [49]. These observations highlight bivalent promoters as regulatory nodes where critical interactions between DNA methylation, H3K27me3 deposition, and transcriptional regulation converge. Our data suggest that DNMT3A plays a direct role in maintaining this balance.

Our hESC models complement patient data and provide a platform for molecular characterization and manipulation. Future studies in differentiated cell types and patient-derived cells will be important to map DNA methylation and gene expression changes in relation to developmental and growth phenotypes. Collectively, our findings reinforce the role of PRC-regulated regions as key methylation targets in growth syndromes and underscore the importance of understanding PRC-DNMT interactions in both normal development and disease.

## Methods

### Generation of hESC mutant lines

The hESC line H1 (WA01) was obtained from WiCell and maintained under feeder-free condition in StemFlex media (Thermo Fisher Scientific) on Cultrex (R&D Systems)-coated plates. Cells were passaged using Accutase (STEMCELL Technologies) and replated in StemFlex medium supplemented with 2 μM thiazovivin (Selleck Chemicals) to enhance viability. To introduce mutations associated with the growth syndromes, synthetic guide RNAs (Supplemental Table 1) (Integrated DNA Technologies) were complexed with recombinant Streptococcus pyogenes Cas9 nuclease (Integrated DNA Technologies) to form ribonucleoprotein (RNP) complexes. These RNPs were delivered into H1 cells by electroporation using either the Neon Transfection System (Thermo Fisher Scientific) or the 4D-Nucleofector (Lonza). DNMT3A frameshift and knockout (KO) cells were generated previously in our laboratory, and their derivation and characterization have been described [50]. For DNMT3A R882H and DNMT3A GoF mutations, a synthetic single-stranded oligonucleotide (ssODN) donor template (Supplemental Table 1) (Integrated DNA Technologies) was co-transfected to facilitate homology-directed repair. Following a 48-h recovery period, transfected H1 cells were replated onto irradiated mouse embryonic fibroblasts (iMEFs) (Thermo Fisher Scientific) to allow for the formation of single-cell-derived colonies. After 10–14 days, individual colonies were picked and genotyped by PCR amplification of genomic regions flanking the target sites, followed by Sanger sequencing to identify clones carrying the desired mutations. At least three clones per mutation category (WT, DNMT3A LoF, DNMT3A GoF, and NSD1 LoF), each derived from separate wells, were subjected to an additional round of single-cell cloning on iMEFs to ensure clonality and eliminate mixed populations prior to downstream applications.

### Real-time quantitative PCR

Total RNA was extracted using Quick-RNA Miniprep Kit (Zymo Research) according to the manufacturer’s protocol, and RNA was reverse-transcribed using High-Capacity cDNA Reverse Transcription Kit (Thermo Fisher Scientific). RT-qPCR reactions were prepared with PowerUp SYBR Green Master Mix (Thermo Fisher Scientific), and amplification was performed on CFX384 Touch Real-Time PCR Detection System (Bio-Rad Laboratories). All reactions were performed in triplicate and melt curve analysis was used to confirm product specificity. DNMT3A (forward: CCTCTTCGTTGGAGGAATGTGC, reverse: GTTTCCGCACATGAGCACCTCA), NSD1 (forward: CAAGGAAGCGAAAACGACAGAGG, reverse: CCGTCCTGTGAGGCATTAGTTC), POU5F1 (forward: CCTGAAGCAGAAGAGGATCACC, reverse: AAAGCGGCAGATGGTCGTTTGG), SOX2 (forward: GCTACAGCATGATGCAGGACCA, reverse: TCTGCGAGCTGGTCATGGAGTT), and 18S ribosomal RNA (RNA18S) as the endogenous control (forward: ACCCGTTGAACCCCATTCGTGA, reverse: GCCTCACTAAACCATCCAATCGG). Relative gene expression levels were calculated using the 2^–ΔCt method, with normalization to RNA18S.

### Western blot

Cells were washed with calcium- and magnesium-free PBS and lysed in 4 × Laemmli sample buffer (Bio-Rad Laboratories) containing 2-mercaptoethanol. Lysates were boiled at 95 °C for 5 min. Protein concentrations were measured using Pierce’s 660 nm Protein Assay Reagent (Thermo Fisher Scientific) according to the manufacturer's instructions. Equal amounts of protein (10 μg) were loaded onto 4–20% Mini-PROTEAN TGX Stain-Free Gels (Bio-Rad Laboratories) and electrophoresed. Proteins were transferred onto PVDF membranes using semi-dry transfer in buffer containing 20% ethanol. Membranes were blocked with EveryBlot Blocking Buffer (Bio-Rad Laboratories) for 10 min at room temperature. Membranes were incubated overnight at 4 °C with a primary antibody against DNMT3A (C-12, Santa Cruz Biotechnology, sc-365769), followed by HRP-conjugated anti-mouse IgG secondary antibody (Cell Signaling Technology, #7076) for 1 h at room temperature. HDAC1 (10E2, Cell Signaling Technology, #59581) was used as a loading control. Between steps, membranes were washed 3 × 5 min with TBST. Immunoreactive bands were visualized using ECL (Thermo Fisher Scientific) and imaged with ChemiDoc imaging system (Bio-Rad Laboratories).

### Flow cytometry

hESCs were dissociated into single-cell suspensions using 0.05% Trypsin–EDTA (Thermo Fisher Scientific) and stained with LIVE/DEAD™ Fixable Dead Cell Stain Sampler Kit (Thermo Fisher Scientific) according to the manufacturer's instructions. Cells were washed with calcium- and magnesium-free PBS supplemented with 5 mM EDTA and 2% FBS and then incubated with APC-conjugated anti-CD326 (EpCAM) monoclonal antibody (clone 323/A3, Thermo Fisher Scientific, MA5-38,715) or mouse IgG1 kappa isotype control antibody at a density of one million cells per ml for 30 min at 4 °C in the dark. After staining, cells were washed and analyzed using NovoCyte Quanteon Flow Cytometer System (Agilent Technologies). Data were acquired and analyzed with FlowJo software (BD Biosciences).

### Mass spectrometry

For sample preparation, culture media was removed, and cells were washed twice with Dulbecco’s Phosphate-Buffered Saline (DPBS). Following DPBS removal, cells were scraped and collected into 1.5 mL microcentrifuge tube, and centrifuged at 2000 g for 3 min. The supernatant was discarded, and the resulting cell pellet was immediately snap-frozen in liquid nitrogen and stored at − 80 °C until further processing. Histones were acid-extracted, derivatized via propionylation, and digested with trypsin, as previously described [51]. Each sample was resuspended in 300 µL of 0.1% FA/mH2O, and 2 µl was injected per run, with 3 technical replicates. Histone extraction, histone modification profiling, and mass spectrometry analysis were conducted at the Mass Spectrometry Technology Access Center at Washington University School of Medicine.

### DNA methylation array and analysis

Genomic DNA was extracted from hESCs using Quick-DNA Kit (Zymo Research). DNA methylation levels were assessed with Infinium MethylationEPIC BeadChip array (Illumina) according to the manufacturer’s instructions. DNA methylation data was processed in R (v4.4.0) based on Bioconductor workflow by Maksimovic and colleagues with minor alterations [52, 53]. In brief, Infinium™ idat files were processed and mapped to human genome version hg38 (GRCh38) using preprocessQuantile in minfi (v1.50.0) [54]. Probes for non-CpG sites, probes not mapped to hg38, and poor quality probes with detection p values greater than 0.01 were removed. Cross reactive probes were removed using maxprobes package (v0.0.2) [55]. For data obtained from patients (Supplemental Table 3), probes with common SNPs at the CpG site or at single nucleotide base extensions were removed (minfi). Sex chromosome probes were removed in patient datasets with males and females. The total number of probes remaining for analysis varied based on the study: GSE299394 (this study), n = 818,992; GSE128801 (TBRS), n = 426,249; GSE191276 (Sotos), n = 792,363; GSE120428 (HESJAS), n = 792,599. M-values and beta-values were then extracted and M-values were adjusted with ComBat for individual BeadChip batch effects if multiple chips were used (sva v3.52.0) [56, 57]. Adjusted M-values were fit to a linear model and tested with an empirical Bayes test with a Benjamini & Hochberg (FDR) correction for multiple comparisons using limma (v3.60.6) [58]. CpG sites with an adjusted p-value < 0.05 were extracted for analyses involving DMPs. Batch corrected M-values were coerced back to beta values by taking the inverse log2 and were reported as methylation percentages. Chromatin state regions were downloaded as bed files and overlapping DMPs were summarized using annotatr (v1.30.0) and genomic ranges (v1.56.2) [59, 60]. DNA methylation plots were generated in R using ggpolot2 (v3.5.1), ggpubr (v0.6.0.999), and ggbreak (v0.1.4) [61–63] or in GraphPad Prism (v10.1.0).

Published datasets were analyzed with the same pipeline as above. The only exception was GSE128801, which was generated using the Infinium Methylation 450 k BeadChip array (Illumina). This dataset was mapped to the human genome version hg19 (GRCh37) and annotated using the corresponding hg19 chromatin state region BED files.

### RELI ChIP-seq enrichment analysis

We collected 10,709 publicly available functional genomic datasets from the Gene Expression Omnibus repository [64] and processed them uniformly using the ENCODE ChIP-seq pipeline [65]. Final ChIP-seq peak sets were indexed by their hg38 genomic coordinates and intersected with the coordinates of DNA methylation sites of interest. To assess the statistical significance of these overlaps, we used RELI [32], which compares observed intersections to a null model. The null model was generated by padding all analyzed CpG sites (818,992 in total) with 100 bp upstream and downstream, then merging overlapping regions to yield 531,738 non-redundant genomic intervals. The distribution of the expected intersection values from the null model resembles, and therefore was modeled as, a normal distribution, where the model parameters were estimated from a series of sampling procedures using RELI. The significance of the observed number of intersections, e.g., a Z-score and the corresponding P-value, was calculated by applying the same padding and merging strategy to DMPs and comparing their overlap with ChIP-seq peaks to the null distribution. As a control for shared HypoMP analysis, we randomly sampled the same number of genomic intervals (n = 6,132) from the null model’s genomic intervals. As a control for all HyperMP and HypoMP mutant analyses, we sampled 13,175 genomic intervals from the null model’s genomic intervals.

### CUT&RUN

CUT&RUN was performed using the CUTANA CUT&RUN Kit (Cat # 14–1048, EpiCypher, NC, USA) and targeted histone mark antibodies (anti-H3K4me3, anti-H3K27me3, EpiCypher, NC, USA; anti-H2AK119ub1, Cell Signaling Technology, MA, USA) following the manufacturer’s protocol. In brief, 0.5 million cells were harvested and incubated with activated Concanavalin A for 10 min at room temperature. For each target histone mark, 0.5 μg of H3K4me3 (Cat # 13-0041), H3K27me3 (Cat # 13-0030), or H2AK119ub1 (Cat # 8240) was added to each sample and incubated overnight at 4 °C. Isotype control (Cat # 13-0042, EpiCypher) was used as a negative control. After overnight incubation, the beads were then washed twice with Cell permeabilization buffer (Wash buffer including 0.01% digitonin), and incubated with protein AG-Micrococcal Nuclease (pAG-MNase) for 1 h at 4 °C. Excessive pAG-MNase was washed out, then chromatin digestion was performed by adding 2 mM CaCl_2_. After chromatin digestion, the stop buffer (Cat # 48,-105, Cell signaling Technology) and 1 ng *E.coli* spike-in DNA (Cat # 18–1401, EpiCypher) were added and incubated for 10 min at 37 °C.

CUT&RUN libraries were prepared with the CUT&RUN library Prep kit (Cat# 14–1001, EpiCypher) following the manufacturer’s instructions. Libraries were quantified with the Quant-iT PicoGreen dsDNA assay kit (Cat# P7589, Thermo Fisher Scientific) and fragment sizes assessed using an Agilent 2100 Bioanalyzer system. Libraries were sequenced to have at least 6 million read pairs using an Illumina NovaSeq 6000. The raw sequencing reads were first trimmed with TrimGalore v0.6.7 and then aligned to the human reference genome hg38 (GRCh38) using Bowtie2 v2.5.1 (parameters –local –very-sensitive-local –no-unal –no-mixed –no-discordant –phred33 –I 10 –X 700). Sam files were sorted and indexed with samtools v1.15.1 to generate bam files, and then duplicate reads were removed using gatk markduplicates (v4.2.6.1). After removing PCR duplicates and unaligned reads, bigWig files were generated from the bam files using bedtools2 v2.30 and reads were normalized by total counts per sample. Heatmap and histone mark enrichment distributions were created using deepTools [66].

### Clustering of H3K4me3-marked regions

H3K4me3 ChIP-seq peaks in H1 hESCs were obtained from ENCODE (ENCSR443YAS). Using the deepTools [66] computeMatrix function in reference-point mode, H3K4me3 and H3K27me3 signals from our CUT&RUN data (GEO accession: GSE301386) were quantified, centered on peak center, and extended from the center ± 10 kb with a bin size of 400 bp. K-means clustering (k = 4) was performed based on the H3K27me3 signal profile across these regions. 

### RNA-seq

Total RNA was extracted from hESCs using Quick-RNA kit (Zymo Research). The quality of RNA was determined by calculating RIN (RNA integrity number) using an Agilent 2100 Bioanalyzer system (Agilent Technologies). RNA-seq libraries were generated using Truseq Stranded Total RNA Gold kit (Illumina) and sequenced on the Illumina NovaSeq X Plus sequencer as 150-bp paired-end reads. RNA-seq data were analyzed using Bioconductor packages Salmon[67], tximeta[68], and DESeq2[69], following the recommended RNA-seq workflow[70]. Genes with a minimum of 10 counts in at least 2 samples were included in differential expression analysis.

## Supplementary Information

Supplemental Figures

Supplemental Table 1.

Supplemental Table 2.

Supplemental Table 3.

Supplemental Table 4.

Supplemental Table 5.

Supplemental Table 6.

## Data Availability

The DNA methylation microarray data have been deposited in GEO under accession number GSE299394. Patient DNA methylation array GEO accession numbers are in Supplemental Table 3. ChIP-seq datasets were obtained as follows: BCOR, KDM2B, PCGF1, and H3K36me2 from GSE104690; H3K36me3 from ENCODE (ENCSR476KTK); RNF2 from GSE105028; and H3K4me3, H3K27me3, and H2AK119ub1 from GSE301386 (this study). RNA-seq data generated in this study have been deposited in GEO under accession number GSE309381.

## Abbreviations

CGI: CpG island
CpG: Cytosine followed by guanine
DMPs: Differentially methylated CpG positions
GoF: Gain-of-function
hESC: Human embryonic stem cell
HESJAS: Heyn-Sproul-Jackson syndrome
HyperMPs: Hypermethylated positions
HypoMPs: Hypomethylated positions
LoF: Loss-of-function
PRC: Polycomb Repressive Complex
PWWP: Pro-Trp-Trp-Pro
RELI: Regulatory Element Locus Intersection
TBRS: Tatton-Brown-Rahman syndrome
