# Supplementary material for: Convergent DNA methylation abnormalities at enhancers and bivalent promoters in human growth disorders
Source: Epigenetics Chromatin. 2025 Dec 27;19:8. doi: 10.1186/s13072-025-00650-1 (PMC12853687; doi:10.1186/s13072-025-00650-1)
Supplement: Supplementary file 1 — Supplemental Figures [file 13072_2025_650_MOESM1_ESM.pdf]

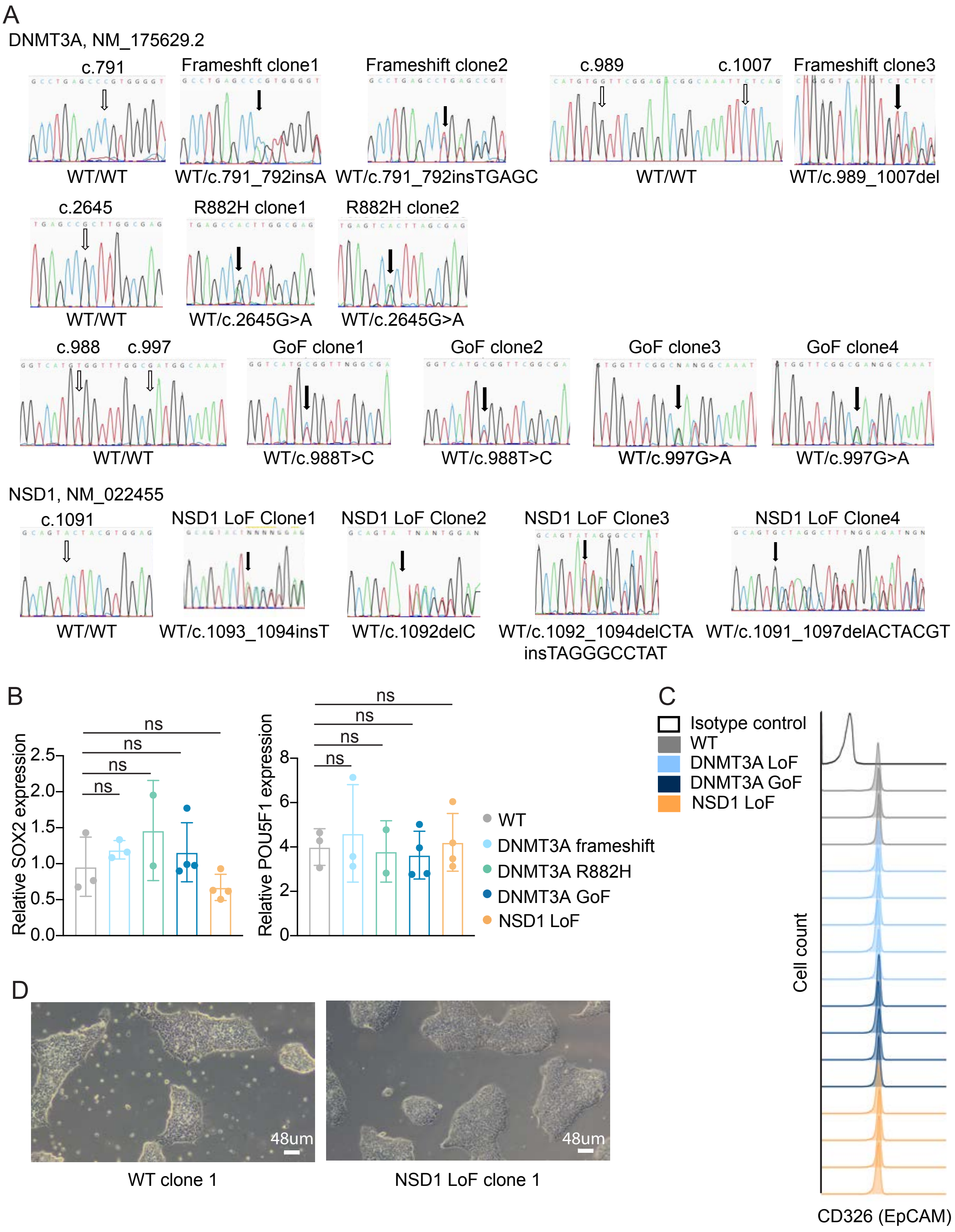

Supplemental Figure 1. **Validation of sequences and pluripotency marker expression of mutant hESCs.** (Related to Figure 1)

(A) Chromatogram traces of mutant clones obtained by Sanger sequencing. Wild-type (WT) sequences are shown as references for each mutation region. White arrows indicate the representative WT positions surrounding the variants, and black arrows mark nucleotide

substitutions, insertions, or deletions in mutant clones. cDNA numbering follows DNMT3A (NM_175629.2) with mutations c.791_792insA, c.791_792insTGAGC, c. 989_1007del (frameshift), c.2645G>A (R882H), c.988T>C and c.997G>A (GoF), as well as NSD1 (NM_022455) with mutations c.1092delC, c.1091_1097delACTACGT, c.1093_1094insT, c.1092_1094delCTAinsTAGGGCCTAT (LoF).

(B) Relative expression of POU5F1 (top) and SOX2 (bottom) transcripts as measured by real-time quantitative PCR, normalized to RNA18S transcript levels. Each dot represents an independent clone.

(C) Flow cytometry analysis of CD326 (EpCAM), a pluripotency marker, in WT and mutant hESC clones. Histograms show the distribution of fluorescence intensity (x-axis, log scale) and the number of cells (y-axis) for each clone.

(D) Representative bright-field images of wild-type (WT) and NSD1 LoF hESC clones cultured under standard feeder-free conditions. All clones, including those not shown, exhibit characteristic pluripotent stem cell morphology with compact colonies and well-defined borders. Scale bar, 48 µm.

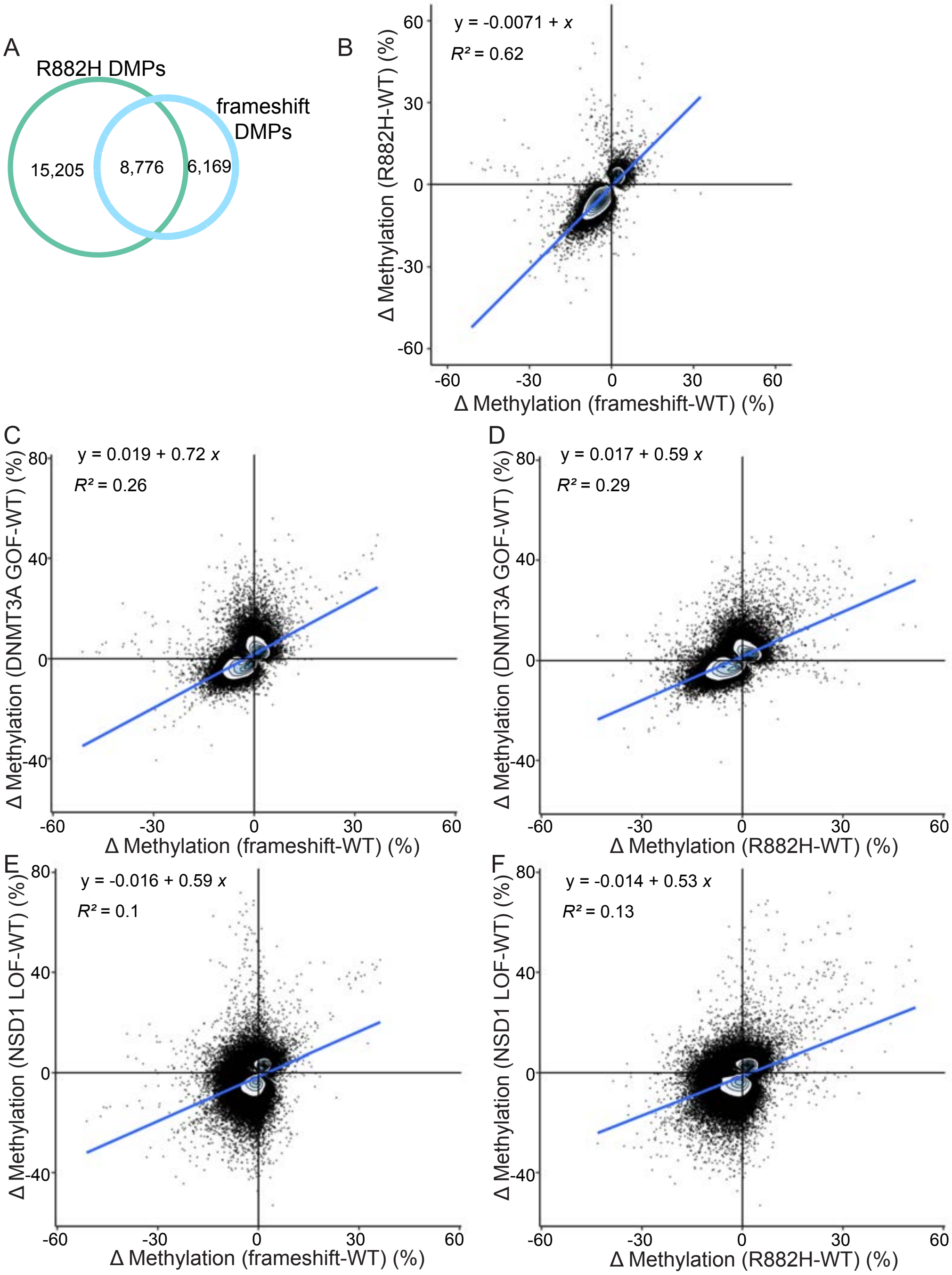

Supplemental Figure 2. Comparison of differentially methylated positions (DMPs) in DNMT3A frameshift and R882H hESC clones. (Related to Figure 2)
(A) Venn Diagram depicting the overlap of DMPs between DNMT3A frameshift and R882H clones. $P < 2.2*10^{-16}$, Fisher's exact test comparing frameshift-unique DMPs to those shared with R882H among all probes. **(B-F)** Pairwise comparison of methylation differences (mutant methylation minus WT methylation) at all DMPs identified in either mutant. Linear regression and coefficient of determination (R^2) are shown. **(B)** DNMT3A frameshift and R882H mutants **(C)** DNMT3A GoF and frameshift mutants **(D)** DNMT3A GoF and R882H mutants **(E)** NSD1 LoF and DNMT3A frameshift mutants **(F)** NSD1 LoF and DNMT3A R882H mutants.

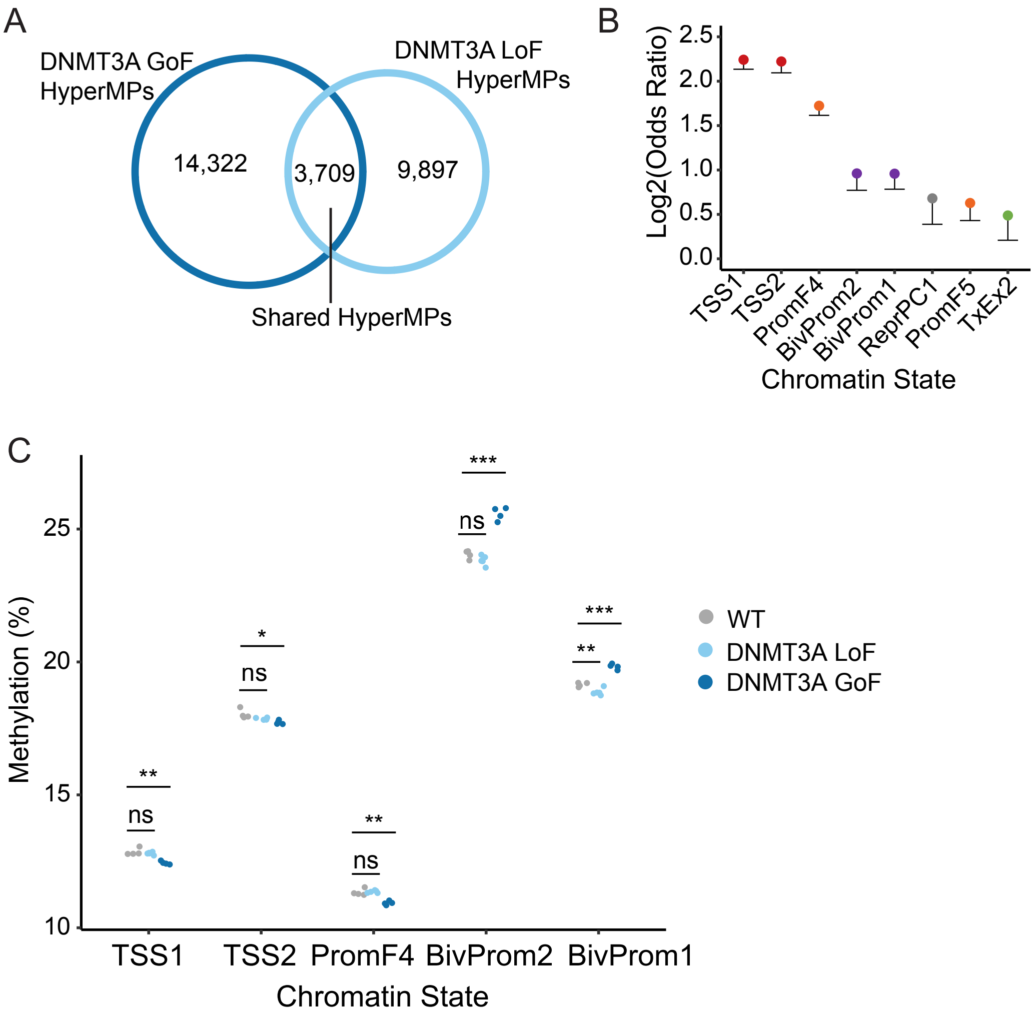

Supplemental Figure 3. Characterization of shared HyperMPs of DNMT3A GoF and LoF mutants. (related to Figure 4) **(A)** Number of overlapping HyperMPs between DNMT3A GoF and LoF mutants. $P<2.2*10^{-16}$, Fisher's exact test of unique DNMT3A GoF in all probes compared to DNMT3A GoF HyperMPs shared with DNMT3A LoF HyperMPs. **(B)** Log_2 odds ratios showing enrichment of shared HyperMPs from DNMT3A mutants across full-stack chromatin states. Dots are colored according to the chromatin state category colors shown in Figure 3 and lower confidence interval bound is shown. Upper confidence intervals were positive infinity and indicate uncertainty in the upper bound of enrichment due to small samples in each state and were left off the graph for clarity. States with significant enrichment ($p<0.05$) are shown. **(C)** Mean DNA methylation values at CpG positions within top five enriched chromatin states. Each dot represents an independent clone. Statistical significance was determined by Student's t-test. *, $p<0.05$; **, $p<0.01$; ***, $p<0.001$; ns, not significant.

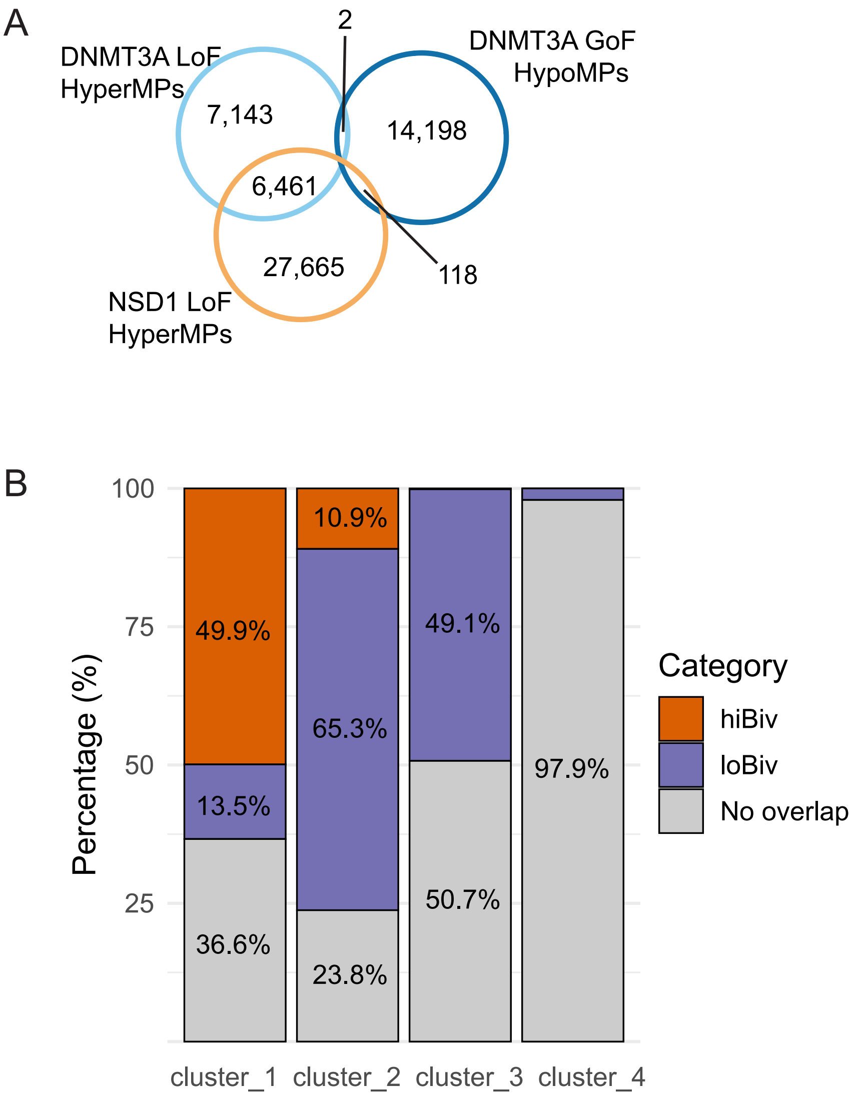

Supplemental Figure 4. (related to Figure 5) **(A)** Venn diagram depicting the overlapping probes between DNMT3A LoF HyperMPs (light blue), DNMT3A GoF HypoMPs (dark blue), and NSD1 LoF HyperMPs (orange). No probes overlap all three groups. **(B)** Overlap between bivalent promoter clusters and Dunican et al. hiBiv/loBiv classifications. Percentage composition of each bivalent promoter cluster showing overlap with high H3K27me3/H3K4me3 ratio (hiBiv, orange) and low H3K27me3/H3K4me3 ratio (loBiv, purple) promoters defined by Dunican et al. (49) in human ESCs, or no overlap (gray). Cluster 1 shows enrichment for hiBiv promoters, while Clusters 2 and 3 show enrichment for loBiv promoters. Cluster 4 shows minimal overlap with either category. Numbers indicate percentage of peaks in each category.
