# Supplementary material for: Convergent DNA methylation abnormalities at enhancers and bivalent promoters in human growth disorders
Source: Epigenetics Chromatin. 2025 Dec 27;19:8. doi: 10.1186/s13072-025-00650-1 (PMC12853687; doi:10.1186/s13072-025-00650-1)
Supplement: Supplementary file 4 — Supplemental Table 3. [file 13072_2025_650_MOESM4_ESM.docx]

| Syndrome | GEO accession | Platform | Samples analyzed |
| --- | --- | --- | --- |
| TBRS | GSE128801 | Infinium Human Methylation 450K BeadChip | GSM3685724, GSM3685725, GSM3685726, GSM3685727, GSM3685728, GSM3685729, GSM3685731 |
| HESJAS | GSE120428 | Infinium MethylationEPIC v1.0 BeadChip | GSM3400677, GSM3400678, GSM3400680 |
| SS | GSE191276 | Infinium MethylationEPIC v1.0 BeadChip | GSM5742874, GSM5742875, GSM5742876, GSM5742877, GSM5742878, GSM5742879, GSM5742880 |

**Supplemental Table 3**. **Growth syndrome patients whose data was reanalyzed for this study.**
